# Effects of imidazoline agents in a rat conditioned place preference model of addiction

**DOI:** 10.1007/s00210-021-02194-z

**Published:** 2022-01-08

**Authors:** V. Şorodoc, G. Rusu-Zota, P. Nechita, C. Moraru, O. M. Manole

**Affiliations:** 1 Department of Internal Medicine (Toxicology), University of Medicine and Pharmacy, “Grigore T. Popa”, 700115 Iasi, Romania; 2 Department of Pharmacology, Clinical Pharmacology and Algesiology, University of Medicine and Pharmacy, “Grigore T. Popa”, 700115 Iasi, Romania; 3 “Socola” Psychiatric Institute, 700282 Iasi, Romania; 4 University of Medicine and Pharmacy, “Grigore T. Popa”, 700115 Iasi, Romania

**Keywords:** Agmatine, Efaroxan, Idazoxan, Tramadol, Conditioned place preference

## Abstract

Agmatine (AG), idazoxan (IDZ), and efaroxan (EFR) are imidazoline receptor ligands with beneficial effects in central nervous system disorders. The present study aimed to evaluate the interaction between AG, IDZ, and EFR with an opiate, tramadol (TR), in a conditioned place preference (CPP) paradigm. In the experiment, we used five groups with 8 adult male Wistar rats each. During the condition session, on days 2, 4, 6, and 8, the rats received the drugs (saline, or TR, or IDZ and TR, or EFR and TR, or AG and TR) and were placed in their least preferred compartment. On days 1, 3, 5, and 7, the rats received saline in the preferred compartment. In the preconditioning, the preferred compartment was determined. In the postconditioning, the preference for one of the compartments was reevaluated. TR increased the time spent in the non-preferred compartment. AG decreased time spent in the TR-paired compartment. EFR, more than IDZ, reduced the time spent in the TR-paired compartment, but without statistical significance. AG reversed the TR-induced CPP, while EFR and IDZ only decreased the time spent in the TR-paired compartment, without statistical significance*.*

**Supplementary Information:**

The online version contains supplementary material available at 10.1007/s00210-021-02194-z.

## Introduction

The imidazoline receptor system is a family of binding sites that recognize compounds with the imidazoline structure. Three main types of imidazoline receptors have been proposed: ${I}_{1}, {I}_{2}$ with subtype ${I}_{2a}$ and ${I}_{2b}$ depending on the affinity for amiloride and ${I}_{3}$ (Bousquet et al. 2020).

The well-studied imidazoline agonist, agmatine (1-amino-4-guanidinobutane, AG) is an endogenous neuromodulator, discovered in the herring semen by Albrecht Kossel in 1910 (Kossel 1910). It is synthesized from l-arginine in a reaction catalyzed by arginine decarboxylase (Gawali et al. 2017; Benitez et al. 2018; Barua et al. 2019) which is found in the mitochondria of the cells in the brain, liver, kidneys, adrenal glands, small intestine, and macrophages (Remko et al. 2017). AG is stored in the granular vesicles in presynaptic terminations and is released into the synaptic cleft after an action potential, where it can act on the receptors or can be degraded (Reis and Regunathan 2000; Raasch et al. 2001; Uzbay 2012). It also acts as an agonist on the imidazoline receptors (Bousquet et al. 2020); GABA_A_ receptors (Neis et al. 2020); and 5-HT2A and 5-HT3 receptors and nicotinic receptors (Benitez et al. 2018), as an antagonist on NMDARs (Benitez et al. 2018; Neis et al. 2020), and inhibits nNOS and iNOS subtypes, but stimulated eNOS subtype (Sharawy et al. 2018; Cigdem et al. 2020). AG is metabolized to either guanidine butyraldehyde and gamma-aminobutyric acid by diamine oxidase or urea and polyamine (putrescine, spermine, spermidine) by the enzyme agmatinase or AG-like protein (Benitez et al. 2018; Barua et al. 2019).

AG has been found in hippocampus, frontal cortex, striatum, locus coeruleus (Barua et al. 2019; Kotagale et al. 2020a, b), amygdala, dorsal raphe nucleus (Selakovic et al. 2019), regions associated with visceral control, neuroendocrine control, pain perception, emotion processing (Reis and Regunathan 2000; Selakovic et al. 2019), cognitive functions, learning, and memory (Kotagale et al. 2020b).

The researcher highlighted the involvement of AG in central nervous system disorders (Neis et al. 2017; Xu et al. 2018). In experimental animal models of Alzheimer’s disease induced by single intracranial amyloid ${\beta }_{1-42}$ peptide injection, AG prevented learning and memory impairment (Dixit et al. 2021). In patients with bipolar disorders, it has been found that there is an increased level of plasma AG in patients with bipolar disorder during a manic episode (Yilmaz et al. 2019) and in patients with first-episode psychosis (Garip et al. 2019). It decreased neuroinflammation and promoted neuroplasticity (Kotagale et al. 2020b). AG exerted antidepressant effects alone (Gawali et al. 2017; Chen et al. 2018; Ostadhadi et al. 2018; Neis et al. 2018) or in combination with other drugs like muscimol, diazepam (Neis et al. 2020), or ketamine (Neis et al. 2018). It also has anti-inflammatory (Neis et al. 2017), anxiolytic (Gawali et al. 2017), and anticonvulsant (Bahremand et al. 2018) properties. In the drug addiction process, it attenuates the symptoms of ethanol (Taksande et al. 2019b), nicotine (Kotagale et al. 2018), and morphine withdrawal (Liu et al. 2018). AG reversed the escalation of intravenous fentanyl self-administration in rats (Morgan et al. 2002), prevented the development of oral fentanyl self-administration in mice (Wade et al. 2008), and inhibited the acquisition and re-acquisition of intravenous morphine self-administration in rats (Su et al. 2009).

The 2-(2,3-dihydro-1,4-benzodioxin-3-yl)-4,5-dihydro-1*H*-imidazole compound, known as IDZ, and the 2-(2-ethyl-3*H*-1-benzofuran-2-yl)-4,5-dihydro-1*H*-imidazole compound, known as EFR, act as an antagonist on ${\propto }_{2}$-adrenergic receptors and as an antagonist on imidazoline receptors, ${I}_{2}$ for the former and ${I}_{1}$ for the latter (Bousquet et al. 2020). Some recent studies have demonstrated the variety of the effects these compounds possess and the involvement of the imidazoline receptor system (Sato et al. 2017; Xuanfei et al. 2017; Chen et al. 2018; Kotagale et al. 2020b;). Both compounds blocked the antidepressant effect of statins and the synergism produced by the coadministration of statins and AG (Rahangdale et al. 2021). Also, in the murine model of Alzheimer induced by administration of ${\beta }_{1-42}$ amyloid peptide, EFR attenuated the effect of AG to reverse memory deficits (Kotagale et al. 2020b). Both IDZ and EFR blocked the antidepressant effect induced by AG (Kotagale et al. 2020a). EFR confirmed the involvement of imidazoline ${I}_{1}$ receptors in the antidepressant effect of AG in models of acute and subacute depression in mice (Chen et al. 2018), in carbophenyline analgesia in neuropathic pain caused by oxaliplatin in mice (Micheli et al. 2020), in maintaining the respiratory drive in newborn rats (Sato et al. 2017) and in the cardioprotective effect exerted by dexmedetomidine against ischemia/reperfusion injury in the spontaneously hypertensive rats (Yoshikawa et al. 2018). EFR stimulated insulin secretion in the MIN6 cell line (Lin et al. 2017), and IDZ reduced hepatic fibrosis in mice (Xuanfei et al. 2017). IDZ confirmed the involvement of imidazoline ${I}_{2}$ receptors, along with ${\propto }_{2}$-adrenergic receptors, in reducing oxidative stress and inflammation in renal function exerted by dexmedetomidine via GSK-3β/Nrf2 in acute kidney injury induced in rats by administration of lipopolysaccharides (Feng et al. 2019). The spontaneous motor activity in rats was decreased by the treatment with IDZ than EFR, more for the former (Rusu et al. 2015). IDZ and EFR reversed the inhibitory influence in ethanol consumption (Taksande et al. 2019b), withdrawal-induced depression (Chimthanawala et al. 2020), and locomotor sensitization (Taksande et al. 2019a) exerted by AG. IDZ was tested in a preliminary double-blind, cross-over, randomized human laboratory study with ten social drinkers included. A single oral dose of 40 mg IDZ reduced the peak blood alcohol level and time to peak compared to placebo. This dose was safe and well tolerated, with a decrease in the systolic blood pressure, but less than 30 mmHg, and it was not considered an adverse effect (Haass-Koffler et al. 2015).

TR is a centrally acting analgesic medication with a synergistic activity as an agonist opioid receptor and a serotonin and norepinephrine reuptake inhibitor (Miotto et al. 2017). This drug is a synthetic codeine analog with an affinity for μ-receptors about ten times lower than codeine and six thousand times lower than morphine (Raffa et al. 1992). The analgesic effect of TR is comparable to that of codeine and ten times lower than morphine (Marquardt et al. 2005).

CPP is an experimental paradigm developed to evaluate the reward in laboratory animals. CPP is a learned behavior based on Pavlovian conditioning. The reinforcer (an unconditioned stimulus) can be a natural reward, like food, or a drug treatment which is administered passively by the experimenter. The reinforcer creates a reward effect and the animals associate this effect with different sensory characteristics from the external environment that become conditioned stimuli. This process depends on learning and memory. The reinforcing effect is manifested by the animal preference for the drug-paired area (Tzschentke 2007; Huston et al. 2013).

As was mentioned above, the imidazoline system is involved in the modulation of cognitive functions and behavior. CPP is the most widely used behavior assay for studying reward or aversion effects of exposure to a drug (Tzschentke 2007). TR is not a substance with high addictive potential (Cicero et al. 1999), but it is widely used for its analgesic properties (Miotto et al. 2017), and has been shown to be addictive in combination with other drugs (Cicero et al. 1999). AG, EFR, and IDZ are drugs that have been tested in combination with other substances with effects on addictive behavior, such as methamphetamine (Thorn et al. 2012), nicotine (Kotagale et al. 2014), and morphine (Wei et al. 2005; Khoshnoodi et al. 2006; Ciubotariu et al. 2011; Ciubotariu and Nechifor 2012), but not with TR. The aim of our study was to investigate the effect of these three imidazoline ligands administration, IDZ, EFR, and AG, in combination with TR in a rat CPP model.

## Materials and methods

### Animals

In the current study, we used 40 adult male white Wistar rats, weighing 350–400 g, procured from the biobase of the “Grigore T. Popa” University of Medicine and Pharmacy Iași. The rats were divided into 5 groups (*n* = 8 animals per group) and were housed in plastic cages under controlled environmental conditions at 21.0 ± 2.0 °C and a 12:12-h light/dark cycle (light on at 07.00 am) with free ad libitum access to food and water (except during the time of the experiments). The animals were allowed to acclimatize for 24 h before they were used in the experiments. All manipulations were carried out between 07.00 and 13.00.

### Drugs

TR, AG, IDZ, and EFR were purchased from Sigma-Aldrich Chemical Co, Germania. All drugs were dissolved in 0.9% NaCl before *ip* administration. Group 1 of animals (coded group 1) was injected with saline 0.9% (0.3 mL/100g *ip*). Group 2 of animals (coded group 2) was injected with TR (40 mg/kg *ip*) and saline 0.9% (0.3 mL/100 g *ip*). Group 3 of animals (coded group 3) was injected with IDZ (3 mg/kg *ip*) and TR (40 mg/kg *ip*). Group 4 of animals (coded group 4) was injected with EFR (1 mg/kg *ip*) and TR (40 mg/kg *ip*). The last one, group 5 of animals (coded group 5), was injected with AG (24 mg/kg *ip*) and TR (40 mg/kg *ip*). The doses of the drugs used were concordant with those applied in other relevant research in the field and previous results from our laboratory (Jackson et al. 1992; Taksande et al. 2010; Rusu et al. 2015; Esquivel-Franco et al. 2018; Rusu-Zota et al. 2019; Rusu-Zota et al. 2021).

### Place conditioning procedure

The CPP apparatus (PanLAB Harvard Apparatus) consisted of two equal-sized plexiglass compartments (25 × 30 × 30 cm), one having black sides and floor and the other having white sides and floor, separated by a grey central area. All the compartments were separated by walls with sliding doors.

The conditioning paradigm consisted of three phases, preconditioning for 1 day, conditioning for 8 days, and postconditioning test on day 10 (Ahsan et al. 2014). The CPP protocol used here is in accordance with previous studies (Sprague et al. 2002; Cha et al. 2014) followed by minor modifications.

On the first day of the experiment (preconditioning), each rat was placed separately into the central grey area for 15 min with free access to all compartments. Placement of the front paws and head was considered as an entry in the compartment. Time spent in each compartment was recorded. The compartment white or black in which animals spent more time was considered the preferred compartment.

The conditioning phase consisted of 8 sessions of 40 min held on consecutive days. On days 2, 4, 6, and 8, the rats received drugs (saline or, TR, or IDZ and TR or, EFR and TR or, AG and TR) and were confined to their least preferred compartment. On days 3, 5, 7, and 9, the rats received saline and were confined to their preferred compartment.

**Fig. 1:**
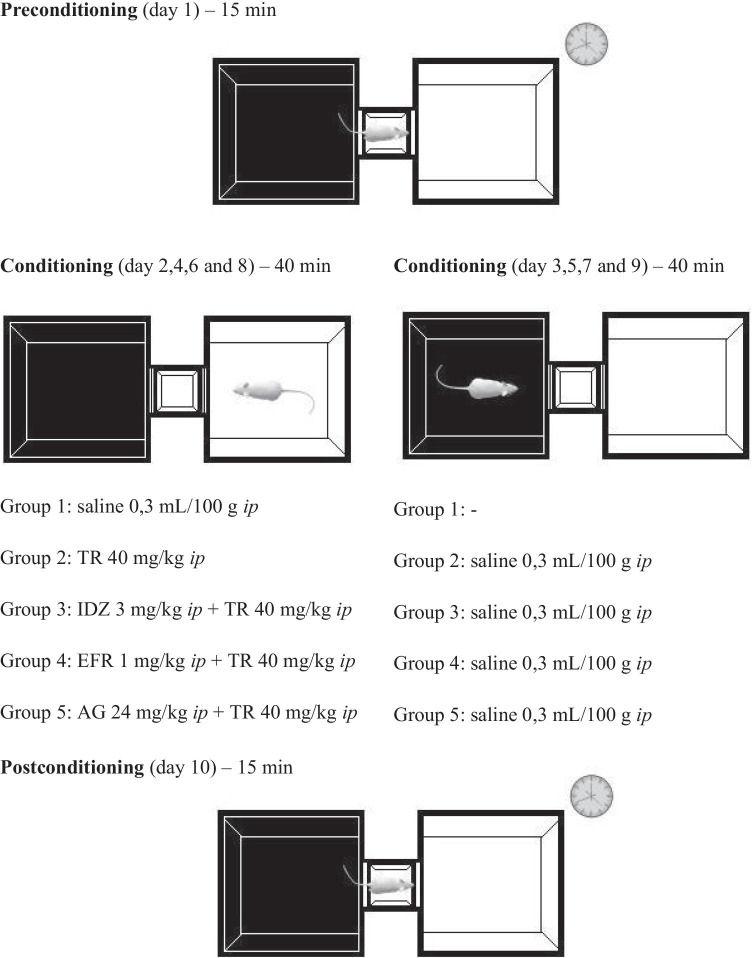
Schematic illustration of the experimental design adopted

On the tenth day of the experiment (postconditioning), each rat in a drug-free state was placed in the central grey area and allowed free access to both compartments for 15 min. Time spent (s) in each compartment was recorded. The difference between the time in the drug-paired compartment during post- and preconditioning was considered as a change in preference score. Figure 1 showed the experimental protocol, doses, and schedule we adopted.

An important consideration in the CPP paradigm is the use of biased/unbiased procedure or biased/unbiased apparatus (Cunningham et al. 2003). In a biased procedure, the assigning of the conditioning stimulus is made basis on the initial preference of subjects as determined in a pre-test, while, in an unbiased procedure, the pairing is done randomly without regard to the initial preference. In a biased apparatus, untrained subjects show a significant preference for one compartment over the other in the absence of conditioning, while, in an unbiased apparatus, the untrained subjects show no preference for one compartment over another (Cunningham et al. 2003; Brielmaier et al. 2008; Prus et al. 2009). In this experiment, we chose to use a biased apparatus and a biased procedure. Some of the drugs (e.g., nicotine) produced CPP more effectively in a biased procedure (Brielmaier et al. 2008). A limitation that can complicate the interpretation of using a biased apparatus or procedure is the “motivational interaction” hypothesis. This may occur because the effect of the drug depends on its interaction with some unconditioned motivational state reflected in the initial cue bias. It has been suggested that by pairing the drug with the initial non-preferred side, the preference shift may develop to the reduction of aversion (stress or fear reduction), rather than a rewarding effect (Cunningham et al. 2003; Le Foll and Goldberg 2005; Brielmaier et al. 2008). However, if the drug is paired with the initial preferred side, a ceiling effect may emerge and prevent detection of CPP (Brielmaier et al. 2008). A disadvantage in using an unbiased procedure is the exclusion of many subjects due to their strong preference for one compartment (Sun et al. 2018). In a conclusion, we chose to use a biased apparatus and procedure to acquire a strong place preference avoiding ceiling effect and not exclude and sacrifice some healthy subjects because it exists an individual vulnerability to drug addiction.

### Ethics approval

All animal procedures and the protocols of the present investigation were approved by the Ethics Committee on Research of the “Grigore T. Popa” University of Medicine and Pharmacy, Iași, România (1/31.10.2013). All procedures complied with the European Communities Council Directive 2010/63/EU. All efforts were made to minimize animal suffering, followed the recommendations of the NIH Guide for the Care and the Use of Laboratory Animals.

### Statistical analysis

The change in preference score was calculated as the difference between time spent in the treatment paired compartment during postconditioning and preconditioning. Each group of experimental animals was characterized as mean change preference (s) ± SEM. Analyses between two groups were conducted using Student᾿s *t*-test and between more than two groups were conducted using a one-way analysis of variances (ANOVA), followed by Tukey post hoc test. A value of *p* < 0.05 was considered significant.

## Results

**Fig. 2:**
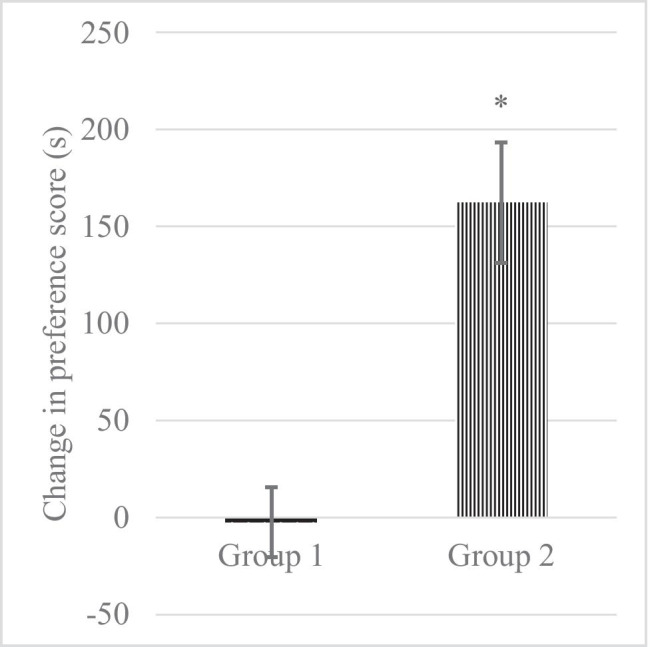
The change in preference score (s) for group 1 treated with saline solution and group 2 treated with TR (*p = 0.0004)

The administration of TR (40 mg/kg *ip*) increased the time the group 2 of rats spent in the drug-paired compartment (162.5 ± 31.091s) as compared to the saline group (− 2.5 ± 17.999 s). Application of Student᾿s *t*-test indicated a significant place preference induced by TR (*p* = 0.0004) as compared to the saline-treated group. Saline treatment in the conditioning compartment did not produce any preference or aversion. Figure 2 illustrated the change in preference score (s) for group 1 and group 2.

**Fig. 3:**
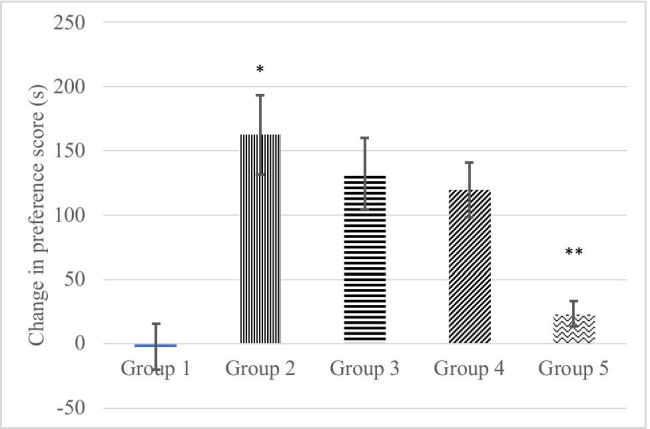
The change in preference score (s) for group 1 treated with saline, group 2 treated with TR (*p = 0.0004 comparative to group 1), group 3 treated with IDZ and TR (*p* = 0.789 comparative to group 2), group 4 treated with EFR and TR (*p* = 0.584 comparative to group 2), and group 5 treated with AG and TR (**p = 0.001 comparative to group 2)

The administration of imidazoline ligands, IDZ (3 mg/kg *ip*), EFR (1 mg/kg *ip*), or AG (24 mg/kg *ip*) on the group 3, group 4, respectively group 5, 15 min prior to the TR treatment during conditioning sessions decreased the time the rats spent in the TR-paired compartment (132.25 ± 28,037 s, 119,625 ± 21,395 s, respectively 22,875 ± 9,833 s). Application of one-way ANOVA showed that imidazoline ligands, IDZ, EFR, or AG, significantly decreased the place preference to the TR-paired compartment (*F*(3,28) = 6.31; *p* = 0.002). The post hoc Tukey test showed that only AG attenuated significantly the TR-induced place preference (*p* = 0.001) as compared to group 2, while IDZ and EFR did not show statistical significance (*p* = 0.789 for group 3, respectively *p* = 0.584 for group 4) as compared to group 2. Figure 3 showed the change in preference score (s) for group 1, group 2, group 3, group 4, and group 5 of experimental animals.

## Discussion

The present investigation studied the interaction between opiate, TR, and imidazoline receptors ligands, AG, IDZ, and EFR, on CPP in rats. Firstly, we demonstrated that the administration of TR at doses to 40 mg/kg *ip* during conditioning induced place preference in rats.

TR is a centrally acting analgesic medication with a synergistic activity as an agonist opioid receptor and serotonin and norephinefrine reuptake inhibitor. Its antinociceptive effect is largely due to its active O-demethylation product metabolite obtained via cytochrome P450 enzyme CYP2D6. The large phenotypic variation of CYP2D6 influences the analgesic potency of a given dose of TR. The risk of TR addiction is slow, but abusive use is found in those with easy access and a history of substance abuse (Miotto et al. 2017).

Several authors have reported that TR can produce CPP in different doses and different routes of administration to mice (Cha et al. 2014; Abdel-Ghany et al. 2015) and rats (Sprague et al. 2002; Rutten et al. 2011; Zhang et al. 2012; Sadeghi-Adl et al. 2020). In the experiment conducted by Abdel-Ghany et al. (2015), administration of 70 mg/kg *sc* of TR increased the time spent in the drug-paired compartment (335s) compared with the time spent in the same compartment before conditioning (135 s) on adult male balb/C mice. Other experimental studies demonstrated that the doses of 18.75 mg/kg, 37.5 mg/kg, 75 mg/kg (Sprague et al. 2002), 6 mg/kg, 18 mg/kg, and 54 mg/kg (Zhang et al. 2012) of *ip* administration of TR produced a significant CPP on adult male Sprague Dawley rats. TR in doses of 2 mg/kg and 4 mg/kg *ip* induced conditioning in CPP task and did not change locomotor activity in preconditioning and postconditioning days on adult male Wistar rats (Sadeghi-Adl et al. 2020). Also, Rutten et al. (2011) showed that tramadol administration (1, 3, and 10 mg/kg *ip*) produced CPP under pain-free conditions on adult male Wistar rats. In the rhesus monkeys’ self-administration model, TR had reinforcing properties (Yanagita 1978).

Other studies showed that TR had not any rewarding or aversive effects in wild-type or MOP knockout mice (Ide et al. 2006) or is not possible to induce tolerance and physical dependence in mice (Miranda and Pinardi 1998).

The CPP paradigm is used to explore the reward properties of a drug in laboratory animals and can also predict the risk of drug abuse in humans (Tzschentke 2007). Morphine (Tahsili-Fahadan et al. 2006; Uskur et al. 2016), ethanol (Sameer et al. 2013), nicotine (Kotagale et al. 2014), cocaine (Davis et al. 2008), and methamphetamine (Thorn et al. 2012) produce CPP and are substances with addictive effects in humans.

The abusive consumption of TR is rare and often seen in patients with a history of substance abuse or used in combination with other drugs (Cicero et al. 1999). TR has a weak addictive potential, but produces reinforcing effects comparable to others opioids. Doses of 37.5 mg/kg and 75 mg/kg of TR-induced CPP are comparable to a single dose of 5 mg/kg morphine (Sprague et al. 2002). Also, Zhang et al. (2012) demonstrated that TR produced CPP dose dependent at the same magnitude like morphine and buprenorphine and was the first who described that sub-effective doses of TR potentiated the sub-effective doses of morphine and buprenorphine to produce CPP.

The region of the brain responsible for the addictive behaviour is named “dopaminergic reward pathway” or “mesocorticolimbic circuit” (Wise 1998). The reward circuit consists of dopaminergic neurons that connect the ventral tegmental area to the nucleus accumbens (Haber and Knutson 2010) and the cerebral cortex (Wise 1998). This pathway is regulated by other brain structures, substantia nigra, ventral striatum, amygdala, hippocampus, lateral habenular nucleus, and raphe nuclei (Haber and Knutson 2010). A drug acts in the ventral tegmental area causing the release of dopamine which, through mesocorticolimbic circuit, releases dopamine from the nucleus accumbens (Wise 1998; Volkow et al. 2019) and can overreact this pathway (Volkow and Morales 2015). It has been found that opioid receptors are expressed in the cortex and limbic system and μ-agonists produce positive reinforcement (Le Merrer et al. 2009). Also, opioids can stimulate the ventral tegmental area and can release dopamine in the nucleus accumbens, producing the sensation of pleasure (Kosten and George 2002). The research showed that TR can release dopamine in the mecorticolimbic circuit (Frink et al. 1996; Sprague et al. 2002; Nakamura et al. 2008) via μ-receptors (Frink et al. 1996; Nakamura et al. 2008).

Secondly, our study revealed that EFR (1 mg/kg) decreased the time spent in the TR-paired compartment in postconditioning more than IDZ (3 mg/kg), but without statistical significance. We have not found any other research paper to investigate the effect of a single administration of IDZ or EFR on TR-induced CPP. Similar results were obtained on morphine CPP in rats. EFR 1 mg/kg reduced its intensity and IDZ 0.25 mg/kg had no influence (Ciubotariu and Nechifor 2012).

EFR binds preferentially to imidazoline ${I}_{1}$ receptors and acts as an antagonist (Bousquet et al. 2020); IDZ is an ${I}_{2}$ imidazoline antagonist, used initially to characterize these receptors (Li 2017; Bousquet et al. 2020), and both compounds act also as an antagonist on ${\propto }_{2}$-adrenergic receptors (Bousquet et al. 2020). The effect of IDZ and EFR on TR-induced CPP could be explained by their action on both imidazoline system and adrenergic system.

It has been proposed that imidazoline receptors, ${\propto }_{2}$-adrenoceptors, NMDARs, and NO level may play a role in modulate reward system activity (Ciubotariu and Nechifor 2012).

The involvement of the imidazoline system in a drug abuse behavior has been intensively studied. The imidazoline compound which received the greatest attention in being evaluated was AG, while IDZ and EFR were used to demonstrate that the effects of AG were via imidazoline receptors (Wei et al. 2005; Su et al. 2008; Thorn et al. 2012; Sameer et al. 2013; Taksande et al. 2019a, b).

The administration of IDZ and EFR prevented the inhibition of ethanol-induced locomotor sensitization by AG in Albino male Swiss mice (Taksande et al. 2019a). EFR reversed the effect of pretreatment with AG (40 mg/kg *ip*) or moxonidine, an imidazoline ${\mathrm{I}}_{1}$ receptor agonist (0.4 mg/kg) or a combination of sub-effective doses of both of them on ethanol (1.25 g/kg *ip*)-induced preference in Swiss albino male mice (Sameer et al. 2013). IDZ counteracted the inhibitory effects exerted by AG on morphine-induced place preference in rats (Wei et al. 2005) and morphine-induced locomotion sensitization (Wei et al. 2007).

The two imidazoline ligand, S23229 and S23230, enantiomers of the S22687 or (5-[2-methyl phenoxy methyl] 1,3-oxazolin-2-yl) amine rose locomotor activity and extracellular dopamine in the rats’ nucleus accumbens. The dopaminergic response after S23229 administration was higher than after S23230 administration, but presented a much lower affinity for ${I}_{1}$ binding sites. The dose of 30 mg/kg of S23229 compound induced a similar dopaminergic and locomotor response to the ones observed after the same dose for cocaine (Barrot et al. 2000).

These results support an important role of imidazoline receptors in mediating effects on drug dependence.

Different studies have demonstrated the complex interaction between α-adrenergic and opioid in the development and expression of opioid dependence (Maldonado 1997). The ${\propto }_{2}$-adrenergic receptors are G protein-coupled and act as inhibitory autoreceptors on noradrenergic neurons. The blocking of ${\propto }_{2}$-adrenergic receptors function by ${\propto }_{2}$-antagonist facilitates noradrenaline transmission. The noradrenaline transmission had an increasing effect on stimulant-induced locomotion activity, a predictor of abuse liability (Schmidt and Weinshenker 2014).

Dexmedetomidine, an ${\propto }_{2}$-adrenergic agonist, administered in doses of 5, 10, or 20 μg/kg *ip* in Wistar albino male adult rats produced CPP with the same intensity of 10 mg/kg morphine (Uskur et al. 2016). The high selective ${\propto }_{2}$-adrenergic agonist, UK 14304 (0.5 mg/kg *ip*) augmented the CPP induced by morphine (0.05 or 0.5 mg/kg *sc*) and the combination of 0.05 mg/kg *sc* morphine and 1 mg/kg *ip* AG, while ${\propto }_{2}$-adrenergic antagonist, yohimbine, and RX821002 attenuated the synergistic effect of morphine and AG (Tahsili-Fahadan et al. 2006). In another study, IDZ abolished the effect of clonidine and AG to decrease the nicotine-induced behavioral sensitization in Swiss albino male mice, using a mechanism mediated by ${\propto }_{2}$-adrenoceptors (Kotagale et al. 2010).

The administration of ${\propto }_{2}$-agonists, clonidine (0.01 or 0.02 or 0.04 mg/kg, *ip*) or tizanidine (0.1 or 0.2 or 0.4 mg/kg, *ip*) or xylozine (2,5 or 5 or 10 mg/kg, *ip*) attenuated the expression of 5 mg/kg *ip* morphine-induced CPP, and this effect was reversed by 0.5 mg/kg, yohimbine, an ${\propto }_{2}$-antagonist, in male NMRI mice (Samini et al. 2008).

In other studies, the administration of 0.05 mg/kg and 0.5 mg/kg of clonidine increased the time spent in the drug-paired compartment in rats through anti-aversive properties more than appetitive properties. While the IDZ (0.5 mg/kg), an ${\propto }_{2}$-adrenergic antagonist, attenuated the effect of clonidine, prazosin, an ${\propto }_{1}$-adrenergic antagonist, did not produce any effect (Cervo et al. 1993).

These results support the involvement of ${\propto }_{2}$-adrenoceptor in opioid dependence.

We demonstrated that EFR, an imidazoline ${I}_{1}$ receptor preferential antagonist, inhibited the acquisition of the TR-induced CPP in rats more than IDZ, suggesting that the effect could be mediated by imidazoline ${I}_{1}$ receptor. A previous study evaluated the role of AG on imidazoline ${I}_{1}$ receptor antisera-selected protein (IRAS), a candidate for imidazoline ${I}_{1}$ receptor, on calcium signal pathway adaptations in morphine dependence. The administration of AG attenuated the increase of intracellular ${Ca}^{2+}$ levels in morphine-dependent CHO-μ/IRAS cells and EFR blocked the inhibitory effect of AG. The CHO-μ/IRAS cell line co-expressed only $\upmu$-opioid receptor and IRAS. These findings support that IRAS, or imidazoline ${I}_{1}$ receptor, has contributions on morphine dependence (Wu et al. 2006).

Thirdly, we showed that the administration of AG (24 mg/kg *ip*) reversed the CPP induced by TR. We have not found any other research papers that evaluated the effects of AG on TR-induced conditioning place preference, but it has been studied in combination with other potentially addictive substances, such as morphine (Wei et al. 2005; Khoshnoodi et al. 2006; Ciubotariu et al. 2011), methamphetamine (Thorn et al. 2012), and nicotine (Kotagale et al. 2014) using CPP paradigm. Other research paper demonstrated that different kinds of doses (0.75 mg/kg or 1.5 mg/kg or 2.5 mg/kg or 10 mg/kg or 20 mg/kg or 40 mg/kg) of agmatine in monotherapy produced neither place preference nor aversion (Wei et al. 2005; Khoshnoodi et al. 2006; Kotagale et al. 2014).

AG inhibited the CPP induced by methamphetamine (Thorn et al. 2012) and nicotine (Kotagale et al. 2014), but on morphine-induced CPP results were contradictory, depending on doses, route, and time of administration and animal used (Wei et al. 2005; Khoshnoodi et al. 2006; Ciubotariu et al. 2011). The doses of 10 mg/kg *ip* or 32 mg/kg *ip* of AG, 10 min before conditioning sessions or in a single administration before postconditioning, decreased the place preference to the methamphetamine (1 mg/kg *ip*) paired compartment on male Wistar Dawley rats (Thorn et al. 2012) and administration of 20 mg/kg *ip* or 40 mg/kg *ip* of AG before conditioning sessions decreased the effect of nicotine (1 mg/kg *ip*) on CPP on mice (Kotagale et al. 2014). On the CPP induced by 3 mg/kg *sc* of morphine on adult male Wistar rats, Wei et al. (2005) demonstrated that different doses of AG (0.75 mg/kg, 2.5 mg/kg, 10 mg/kg, or 40 mg/kg) in *sc* administration 30 min before conditioning sessions inhibited the acquisition of CPP, while Ciubotariu et al. (2011) demonstrated that 2 mg/kg or 4 mg/kg of AG in *ip* administration before conditioning sessions did not exert any effect. By contrast, the experiment conducted by Khoshnoodi et al. (2006), administration of AG (1 mg/kg, 5 mg/kg, or 10 mg/kg, *ip*) during the conditioning sessions enhanced the effect of various non-effective doses of morphine (0.01 mg/kg, 0.05 mg/kg, 0.1 mg/kg, or 0.5 mg/kg, *sc*) on producing CPP on male NMRI mice.

Also, AG inhibited intravenous self-administration of morphine in male Sprague Dawley rats in intragastric administration (Su et al. 2009) and inhibited the development of morphine dependence in male Wistar rats (Liu et al. 2018). Pretreatment with 40–80 mg/kg *ig* AG administered at the beginning of the use of morphine inhibited the acquisition of 2 mg/kg *sc* morphine-induced discrimination and chronic administration of 40–80 mg/kg *ig* AG attenuated morphine-associated discrimination, a paradigm used to study subjective effects of drugs abuse in humans (Su et al. 2008). AG (10 mg/kg *sc*) inhibited morphine-induced locomotion sensitization and reversed the increase of striatal extracellular 3,4-dihydroxyphenylacetic acid and homovanillic acid levels after 3 days of morphine withdrawal (Wei et al. 2007).

The proposed mechanisms for the inhibitory effect of AG on drug-induced conditioning are numerous.

AG in doses of 20 mg/kg and 40 mg/kg decreased the ethanol consumption when it was being directly delivered in the right posterior ventral tegmentum area in the operant conditioning paradigm on Wistar rats. The same doses of AG and drugs are known to raise its endogenous levels like l-arginine, aminoguanidine, and arcaine, attenuated the ethanol consumption in a two-bottle choice paradigm on Wistar rats. The effect of AG on ethanol intake was potentiated by moxonidine (${I}_{1}$ agonist) and 2-BFI (${I}_{2}$ agonist) and blocked by IDZ (${I}_{2}$ antagonist) and EFR (${I}_{1}$ antagonist), suggesting the implication of imidazoline receptors in the regulation of brain dopaminergic signaling in the ventral tegmentum area (Taksande et al. 2019a, b). AG attenuated the development of morphine physical dependence, regulated long-term reward memory, and inhibited the expression of the transcription factor FosB that is a physical dependence indicator. These effects were reversed by IDZ, and not by yohimbine, suggesting also the implication of imidazoline receptors (Wei et al. 2005).

Alterations in hippocampus neurogenesis have been shown to play an important role in drug addiction and relapse. A dose of 10 mg/kg of AG inhibited the development of morphine dependence in male Wistar rats and counteracted the effects of morphine to decrease the proliferation of hippocampal neural progenitors in the granule cell layer and the levels of hippocampal cAMP, pCREB, and BDNF (Liu et al. 2018). AG activated the 5-HT1A receptors involved in hippocampus neurogenesis and influenced hippocampal neuroplasticity by increasing cell proliferation and dendritic complexity (Olescowicz et al. 2018). Also, in an experimental model of Parkinson’s disease induced by administration of rotenone in adult Sprague Dawley rats, AG prevented the loss of dopaminergic neurons in the stratum by increasing the cellular defense mechanism against oxidative injury and the level of neurotrophic factors (Bilge et al. 2020). These studies showed that AG can influence the addictive potential of a substance by modulating neurogenesis.

AG, an imidazole ${I}_{1}$ and ${I}_{2}$ agonist receptors, can increase the monoamine levels (serotonin, dopamine, and norepinephrine) (Olescowicz et al. 2018) and the monoamine oxidases, MAO-A and MAO-B, which are enzymes that inactivate the neurotransmitters, bind allosteric to ${I}_{2}$ receptors (Bektas et al. 2015).

Khoshnoodi et al. (2006) showed that AG potentiated the morphine-induced CPP by modulating NO levels. In another study, while AG did not exert any effect, 0.4 mmol/kg of zinc chloride, a modulator for NO levels, decreased the morphine-induced CPP, supporting the influence of NO in addictive properties of drugs (Ciubotariu et al. 2011).

## Conclusions

In conclusion, the present study aims to evaluate on CPP test the interaction between imidazoline receptors ligands, AG, IDZ, EFR, and an opiate, TR. We showed that administration of TR increased the time spent in the drug-paired compartment and produced CPP. AG significantly blocked the acquisition of TR-induced CPP, while IDZ and EFR decreased, more for EFR, the time spent in TR-paired compartment, but without statistical significance.

These results suggest that AG influences the behavioral effects of TR, but because of its affinity for multiple receptors and various physiological functions, the exact mechanism is not clear. More than that, it is possible a different involvement of the types, ${I}_{1}$ and ${I}_{2}$, of imidazoline receptors, due to the fact that EFR, an ${I}_{1}$ imidazoline antagonist, reduced the intensity of the TR effects, more than IDZ, an ${I}_{2}$ imidazoline antagonist.

Imidazoline receptors are involved in multiple physiological and patho-physiological processes throughout the body. The connections between the imidazoline system and other neurotransmitter systems in the brain suggest the complexity of psycho-pathological changes present in certain nervous system diseases such as impairment of cognitive functions, changes in behavior, and stress activity.

## Supplementary Information

Below is the link to the electronic supplementary material.

Supplementary file1 (XLSX 34 KB)

## Data Availability

Not applicable.

## Abbreviations

5-HT1A: 5-Hydroxytryptamin 1A receptor
5-HT2A: 5-Hydroxytryptamin 2A receptor
5-HT_3_: 5-Hydroxytryptamin 3 receptor
AG: Agmatine
BDNF: Brain-derived neurotrophic factor
cAMP: Cyclic adenosine monophosphate
CHO-μ/IRAS: Mu opioid receptor/imidazoline receptor antisera-selected
CPP: Conditioned place preference
CYP2D6: Cytochrome P450 2D6
EFR: Efaroxan
eNOS: Endothelial nitric oxide synthase
GABA A: γ-Aminobutyric acid type A receptor
GABA_A_: γ-Aminobutyric acid type A
GSK-3β/Nrf2: Glycogen synthase kinase-3β (GSK-3β)/nuclear factor erythroid 2-related factor 2 (Nrf2)
IDZ: Idazoxan
ig: Intragastric
iNOS: Inducible nitric oxide synthase
ip: Intraperitoneal
IRAS: Imidazoline receptor antisera-selected
MAO-A: Monoamine oxidase A
MAO-B: Monoamine oxidase B
MOP: μ-Opioid receptor
NMDARs: N-Methyl-d-aspartate receptors
nNOS: Neuronal nitric oxide synthase
NO: Nitric oxide
pCREB: cAMP response element-binding protein
sc: Subcutaneous
TR: Tramadol
